# Patients’ and physicians’ awareness of clinical symptoms and disease severity in tuberous sclerosis complex

**DOI:** 10.1186/s13023-024-03118-9

**Published:** 2024-03-08

**Authors:** Matthias Sauter, Lea Weber, Dominik Jung, Michael Weremko, Dorothea Bachmann, Michael Fischereder, Hagen Sjard Bachmann

**Affiliations:** 1 Klinikverbund Allgäu, Robert-Weixler-Str. 50, 87439 Kempten, Germany; 2 Institute of Pharmacology and Toxicology, Centre for Biomedical Education and Research (ZBAF), School of Medicine, Witten/Herdecke University, Witten, Germany; 3 Institute of Pharmacogenetics, University Hospital Essen, University of Duisburg-Essen, Essen, Germany; 4 Tuberöse Sklerose Zentrum, Klinikum Der Universität München, Ziemssenstr. 5, 80336 Munich, Germany

**Keywords:** Tuberous sclerosis complex, Disease severity, Surveillance, Disease awareness, Healthcare

## Abstract

Tuberous sclerosis complex (TSC) is a rare inherited disease with the potential to affect virtually every organ system. Clinical presentation is age- and partly sex-dependent and varies broadly with respect to disease manifestations including treatment-refractory epilepsy, intellectual disability and TSC-associated neuropsychiatric disorders, chronic kidney disease or progressive lung function decline. Given the complexity of this disease, multidisciplinary care in specialized TSC centres is recommended. We aimed to elucidate the state of knowledge of patients/caregivers and physicians on individual disease manifestations. We further examined whether the association to a TSC centre has an impact on the comprehensive consideration of potential disease manifestations. Therefore, a survey was performed in a cohort of German TSC patients and their physicians. Complete information was available for 94 patients with a median age of 18 years [range 1–55] and a sex distribution of 53.2% (male): 48.8% (female). Using almost identical questionnaires for patients/caregivers and their respective physician, there was a good correlation for disease assessments associated with relevant morbidity and mortality like epilepsy, renal angiomyolipoma, cardiac rhabdomyomas or intellectual disability. Correlation was moderate for several neuropsychiatric disorders and only poor for hypomelanotic macules, dental pits or retinal achromic patches. Estimation of overall disease severity using a numeric rating scale correlated highly significantly (Pearson correlation coefficient = 0.767; *p* < 0.001) between patients/caregivers and physicians. In general, physicians more likely quoted items as ‘unknown’ than patients (822 answers vs. 435 answers in the respective groups). Questionnaires completed by physicians who were associated with a specialized TSC centre declared a significantly lower proportion of items as unknown (mean 8.7% vs. 20.5%; *p* < 0.001). These findings indicate that patients treated by specialized TSC centres seem to obtain a more comprehensive surveillance. Furthermore, it shows that there were reasonable surveillance strategies in general and sufficient patient/caregiver interaction and education in the examined cohort. However, for the most prominent disease characteristics there was a good awareness within both the patients/caregivers and the physicians group.

**Supplementary Information:**

The online version contains supplementary material available at 10.1186/s13023-024-03118-9.

## Introduction

Tuberous sclerosis Complex (TSC) is a rare autosomal-dominantly inherited disorder affecting one in 6000 newborn [1]. Mutations in one of two tumor suppressor genes lead to an overexpression of the mechanistic (formerly: mammalian) target of rapamycin (mTOR) complex 1 [2]. This results in hamartomatous tumor lesions in a wide variety of organs and can be fatal [3]. Typical organs affected are the heart, the kidneys, the skin, and the retina, as well as the central nervous system. Reasons for disease-specific mortality are as diverse as the disease itself: TSC kidney disease was found to lead to most deaths, apart from this sudden death in epilepsy (SUDEP), pulmonary lymphangioleiomyomatosis (LAM) and subependymal giant cell astrocytoma (SEGA) occur as well [4, 5].

Clinical presentation is highly variable and may even differ markedly within families. Furthermore, several manifestations are age dependent, like cardiac rhabdomyoma (early infancy) or pulmonary LAM (adulthood, predominantly female sex [3]). About 80% of TSC patients have a history of seizures that often are difficult to treat or even refractory to treatment [6]. Furthermore, a population-based study found intellectual disabilities in approximately 50% of affected individuals; as many as 30% were classified as profoundly disabled [7].

Treatment strategies are based on the individual disease characteristics and are therefore highly diverse, including, e.g. antiseizure medication as well as tumor surgeries or treatments with mTOR inhibitors [8, 9].

State-of-the art care requires a multidisciplinary team and includes life-long surveillance as well as treatment of relevant disease manifestations considering the individual burden of disease [8, 10, 11].

Although specialized TSC centres likely ameliorate patient care and disease outcome, as far as we know, this has not been evaluated systematically. Furthermore, it seems to be important that the patients or their caregivers are informed about the particular disease manifestations that allow them to actively contribute to a comprehensive medical concept [12]. So far published data on disease manifestations in TSC cohorts derive from the physician’s point of view, often neglecting the patient’s/caregiver’s knowledge about individual disease manifestations.

We therefore aimed to elucidate the concordance between patient’s and physician’s knowledge of individual disease characteristics. Furthermore, we aimed to investigate whether the responsible physician’s affiliation to a specialized TSC centres has an impact on the background knowledge about the disease and finally, we wanted to correlate the perception of individual disease severity between patients/caregivers and their physicians.

## Methods

### Questionnaires

Two questionnaires were designed in German with almost identical content, one for patients or their caregivers and one for their physicians. Both asked for well-known manifestations of TSC including major and minor diagnostic criteria. Possible answers included’presence’/’absence’ or’unknown’ for the particular manifestation. In addition, we asked for a global rating of disease severity using a numeric rating scale with’1’ being the lowest and’10’ the highest imaginable degree of disease severity. To reduce the effort for participating physicians, only the patient questionnaire collected information on demographic data, ECOG (Eastern Cooperative Oncology Group) performance scale [13], Barthel ADL index [14] and a certified degree of disability (German social security system: “Grad der Behinderung”, range 0–100%). The questionnaires were distributed in 2013 via the German Tuberous Sclerosis patient organization (Tuberöse Sklerose Deutschland e.V.) to their members. The recipients were asked to forward the appropriate questionnaire to their physicians. Detailed study information in written form was provided with the opportunity to contact the investigators for further information. Participants or their legal representatives (in case of children or intellectual disability) gave written informed consent for the enrolment in the study. The study was conducted in accordance with the principles of the Declaration of Helsinki and approved by the local ethics committee (University Hospital Essen 12-5107-BO).

### Statistics

Statistical analysis was performed using IBM SPSS Statistics version 28. Calculations included crosstabs, Spearman’s rank order correlation and Mann–Whitney U test. A *p*-value < 0.05 was considered statistically significant. Cohen’s kappa coefficient was used to measure the inter-rater reliability for the particular items with application of the interpretation from McHugh [15].

The correlation of patient´s rating of disease severity with Barthel index, degree of disability and ECOG performance status was evaluated via Pearson correlation coefficient (PCC). Data are shown in Additional file 1: Table S4.

## Results

### Participants

From over 750 questionnaires sent out to patients and physicians, 129 completed patient questionnaires and 101 physician questionnaires were received back. This corresponds to a response rate of approximately 15.3% (Additional file 1: Fig. S1).

A complete set of a patient and a physician questionnaire was available for 95 patients, with information on the physicians’ affiliation being available for 94 cases. Consequently, all analyses were conducted based on these 94 matched cases. Relevant patient characteristics including the prevalence of TSC manifestations are shown in Table 1.

**Table 1:** Demographic data and prevalence of maindisease manifestations in the respective groups

	Patient questionnaire	Physician questionnaire
Number	94	
Sex	Male: 50 (53.2%), Female: 44 (46.8%)	
Age	Median: 18 years [range 1–55]	
Manifestations		
*Skin*		
Hyomelanotic macules	84 (89.36%)	70 (74.47%)
Facial angiofibroma	80 (85.11%)	66 (70.21%)
Fibrous cephalic plaque	37 (39.36%)	25 (26.6%)
Chagrin patch	45 (47.87%)	32 (34.04%)
Periungual fibroma	43 (45.74%)	28 (29.79%)
Dental pits	36 (38.3%)	23 (24.47%)
*Eye*		
Retinal hamartoma	23 (24.47%)	18 (19.15%)
Retinal achromatic patch	6 (6.38%)	3 (3.19%)
*Brain*		
SEN	74 (78.72%)	66 (70.21%)
SEGA	37 (39.36%)	39 (41.49%)
Tubera	66 (70.21%)	74 (78.72%)
Epilepsy	84 (89.36%)	80 (85.11%)
Infantile spasms	53 (56.38%)	42 (44.68%)
*Neuropsychiatric disorders*		
Autism/autism spectrum disorder	41 (43.62%)	41 (43.62%)
Aggressive behaviour	32 (34.04%)	26 (27.66%)
Anxiety	31 (32.98%)	25 (26.6%)
Compulsion	33 (35.11%)	17 (18.09%)
Intellectual disability	58 (61.7%)	57 (60.64%)
Non-verbal communication	16 (17.02%)	16 (17.02%)
Unable to communicate	10 (10.64%)	5 (5.32%)
*Heart*		
Cardiac rhabdomyoma	52 (55.32%)	50 (53.19%)
*Kidney*		
Renal angiomyolipoma	54 (57.45%)	54 (57.45%)
Angiomyolipoma hemorrhage	9 (9.57%)	5 (5.32%)
Renal insufficiency	12 (12.77%)	6 (6.38%)
*Lung*		
LAM	10 (10.64%)	14 (14.89%)
Pneumothorax	6 (6.38%)	3 (3.19%)
*Locomotor system*		
Scoliosis	24 (25.53%)	17 (18.09%)
Impaired ambulatory ability	31 (32.98%)	19 (20.21%)
Unable to walk	5 (5.32%)	5 (5.32%)

A more detailed table is given in Additional file 1: Table S1

Data are displayed in number and percentage of the relevant cohort (in parenthesis) if not otherwise declared

SEN, subependymale nodule; SEGA, subependymale giant cell astrocytoma; LAM, pulmonary lymphangioleiomyomatosis

**Fig. 1:**
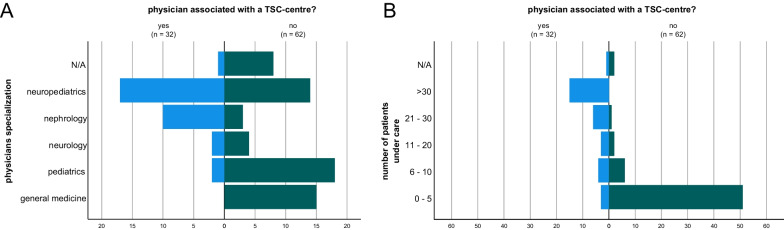
Information on the physicians who participated in the study and their TSC centre association. **A** Overview of the respective specialization of the physicians and their association with a TSC clinic. **B** Information on the TSC centre association as well as the number of patients to be cared for

The participating physicians’ area of expertise was neuropediatrics (34%), pediatrics (20%), partially with specialization in pediatric nephrology, cardiology or pneumology, general medicine (16%), nephrology (14%) and neurology (6%) (Fig. 1A). 10% of respondents did not provide information on their specialization. 32 (34%) were members of a certified TSC centre whereas 62 (65%) were not. On average, physicians affiliated to a TSC centre cared for substantially more TSC patients than physicians not affiliated to a TSC centre (Fig. 1B), e.g., 15 physicians affiliated to a TSC centre cared for more than 30 patients, while no physician not affiliated to a TSC centre cared for more than 30 patients.

### Correlation between patients’ and physicians’ estimation on disease characteristics

**Fig. 2:**
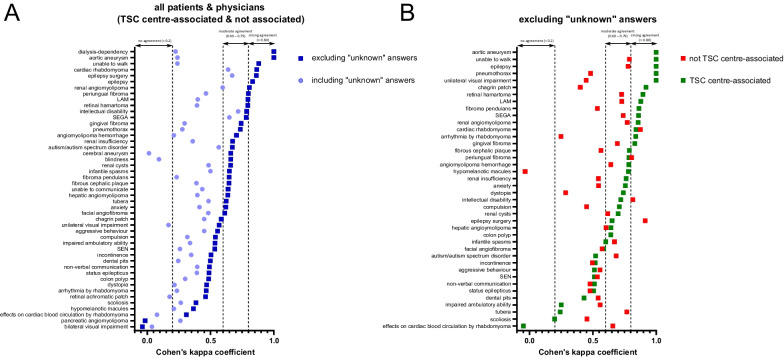
Cohen’s kappa coefficient showing the correlation between patient and physician questionnaires concerning the estimation of different disease manifestations. **A** Plot of Cohen’s kappa coefficient with (light blue) and without (dark blue) response option ‘unknown’, based on all patients and physicians’ questionnaires. **B** Plot of Cohen’s kappa coefficient based on ‘yes’ or ‘no’ responses, divided into physicians with TSC centre association (green) and without TSC centre association (red)

First, we aimed to analyze the effect of association with a TSC centre on the estimation of different disease characteristics using Cohen’s kappa coefficient. Aortic aneurysm, epilepsy, renal angiomyolipoma and cardiac rhabdomyoma, which are among the most severe symptoms of TSC, exhibited “strong” correlation between patient and physician questionnaires when disregarding ‘unknown’ answers (Fig. 2A), especially in the group of TSC centre-associated physicians (Fig. 2B). Several other symptoms and characteristics, like gingival fibroma, history of angiomyolipoma hemorrhage or pneumothorax showed moderate agreement when excluding ‘unknown’ answers (Fig. 2A). Of note, history of pneumothorax showed a clear difference regarding the association to a TSC centre (Fig. 2B). For hypomelanotic macules, moderate to strong correlation was found in the TSC centre-associated group, but no correlation was found in the group not associated with a TSC centre when considering only’yes’/’no’ answers (Fig. 2B).

By including the’unknown’ answers, substantial correlation could be found for SEGA, epilepsy, any neuropsychological problems, intellectual disability, renal angiomyolipoma, and cardiac rhabdomyoma (Fig. 2A). No agreement was found for cerebral aneurysm and blindness due to the high number of ‘unknown’ answers for these symptoms (Fig. 2A). The complete data set is shown in Additional file 1: Table S2.

The frequency of items declared as ‘unknown’ for the physician and the patient group is given in Additional file 1: Table S3. Briefly, the presence of disease-associated retinal pathologies was frequently declared as’unknown’ in both groups. In the physician group the most frequent item declared as’unknown’ was dental pits (n = 36) and some skin manifestations (periungual fibroma, chagrin patch, hypomelanotic macule, fibrous cephalic plaque) were frequently stated as ´unknown´ as well. Following the above findings of a high correlation for epilepsy in both groups, no response was declared as’unknown’ for this manifestation in either group. In general, physicians quoted items as’unknown’ more frequently than patients (822 answers vs. 435 answers in the respective groups).

However, when differentiating between physicians associated with a TSC centre and those who are not, only the latter reported a significantly higher proportion of items as’unknown’ compared to patients (Kruskal–Wallis test *p* < 0.0001; post-test *p* < 0.0001 (TSC clinic) vs. *p* > 0.999 (no TSC clinic); (Additional file 1: Table S3)).

Questionnaires completed by neuropediatricians had a substantially lower number of manifestations declared as’unknown’ compared to general or otherwise specialized pediatricians (mean 7.97% vs. 18.21%; Additional file 1: Table S4). Nephrologists declared fewer items as’unknown’ compared to general practitioners (mean 12.48% vs. 23.65%) or neurologists (mean 12.48% vs. 44.34%).

### Numeric rating scale of disease severity

**Fig. 3:**
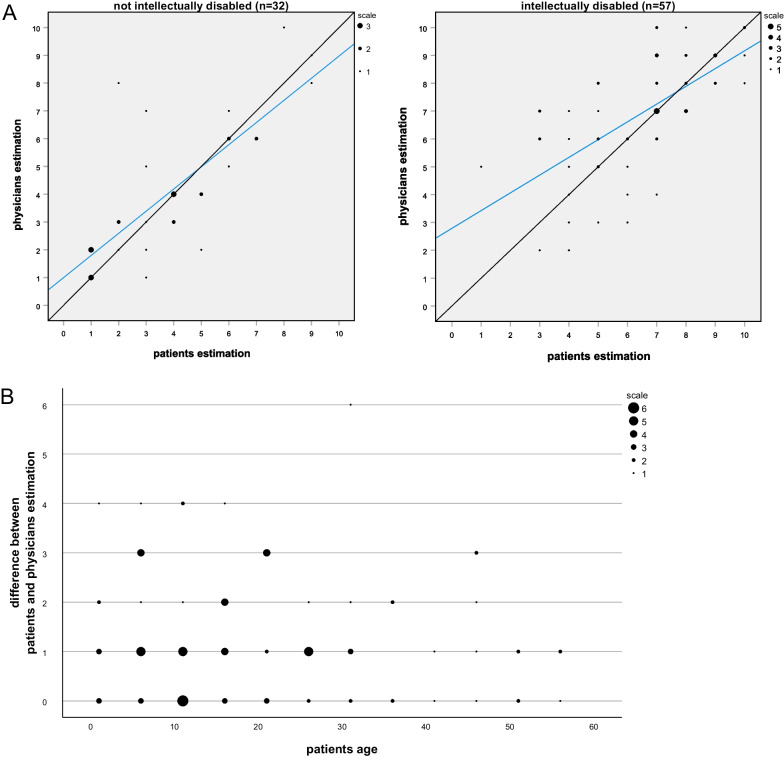
Correlation of global disease severity estimations. The degree of disease severity was estimated using a numeric rating scale (1 = lowest and 10 = highest imaginable degree of disease severity) for the corresponding 89 physician and patient questionnaires and the comparisons were stratified by intellectual disability. **A** Correlation of global disease severity estimation on the degree of disability. Information on disease severity was not available for 4 paired questionnaires, information on intellectual disability was not available for 1 questionnaire. The diagonal lines indicate perfect matches between physicians and patient, the blue lines represent linear trend lines. **B** Differences between estimations of patients and physicians plotted against the patients’ age. Dot size indicates the number of hits for the respective combination

Estimation of overall disease severity using a numeric rating scale correlated highly significant (PCC = 0.767; *p* < 0.001) between patients and physicians as indicated in Fig. 3. There was a discrepancy of at least four points in only five out of 90 valid pairs (5.5%). Interestingly, the physicians declared the disease severity as higher in all of these cases. Four of the five patients with marked discrepancy in rating were < 18 years whereas the percentage of participants < 18 years in the whole cohort was 48% (45 out of 94). All these four children had epilepsy; however, the evaluation of neuropsychological problems and autism differed in some patients. Unsurprisingly, patient’s rating of disease severity correlated negatively with Barthel index (PCC = − 0.643; *p* < 0.001) and positively with the degree of disability (PCC = 0.570; *p* < 0.001) as well as ECOG performance status (PCC = 0.692; *p* < 0.001). For further information see Additional file 1: Table S5.

## Discussion

In this study, patients/caregivers affected by TSC, and their respective physicians were asked to complete a questionnaire to assess their knowledge of the many different disease manifestations of TSC. Furthermore, we aimed to investigate whether affiliation with a specialized TSC centre influences the physician’s knowledge of diverse features of the disease, likely to result in a more comprehensive surveillance. To our knowledge, this is the first study on TSC with this attempt. In general, we observed good awareness for the most prominent manifestations of the disease within both recipients’ groups.

The percentages of disease manifestations in our cohort are comparable to findings from other cohorts. Comparing the symptoms reported from physicians with baseline data from the TOSCA study, the largest registry study on TSC patients to date, percentages are almost identical for epilepsy (85.1% vs. TOSCA: 83.5%), tubera/focal dysplasias (78.8%TOSCA: 82.2%) and subependymal nodules (SEN) (70.2% vs. TOSCA: 78.2%) [16]. As TOSCA data retrieval was based on physician’s reporting only [17], it appears more accurate to compare the physicians’ findings from our study rather than the patient reports. However, there were some relevant discrepancies: Cutaneous manifestations like facial angiofibroma (70.2% vs. TOSCA: 57.3%), fibrous cephalic plaque (26.6% vs. TOSCA: 14.1%) or periungual fibromas (29.8% vs. TOSCA: 16.7%) were more frequent in this study as were dental pits (24.5% vs. TOSCA: 4.7%) and the renal symptoms angiomyolipoma (57.5% vs. TOSCA: 47.2%) and cysts (37.2% vs. TOSCA: 22.8%). These differences may be partly explained by differences in the participants’ age. Patients in our study were older (median 18 years) than those in the TOSCA study (median 13 years) [16] and the aforementioned symptoms typically occur in a higher percentage of older patients [3, 18]. However, some manifestations typically seen in young patients were more frequent in our study, such as hypomelanotic macules (74.5% vs. TOSCA: 66.8%) and cardiac rhabdomyomas (53.2% vs. TOSCA: 34.3%) [3].

Differences in the presence of a subependymal giant cell astrocytoma (SEGA) which is quite high in our study (41.5% vs. TOSCA: 24.4%) may be partly explained by the lack of a commonly accepted definition of SEGA. The higher presence of retinal hamartoma (19.2% vs. TOSCA: 14.0%) might be due to a more comprehensive examination of patients in our national study compared to the examination of the patients in the TOSCA study, including patients from different countries (Asia, Eurasian, Africa), some with a less developed health care system.

Furthermore, the frequency of disease manifestations found in our study fairly agree with recently published data from Zöllner et al., examining costs and cost-driving factors in TSC affected children, adolescents [19] and adults [20] respectively. The information in these studies derived from patients/caregivers as well. Given the fact that we report data from a cohort including children as well as adults, percentages of typically age dependent manifestations were in between the respectively reported rates as for example renal angiomyolipoma (57.5% vs. children/adolescents 45.4% and adults 59.4%), cardiac rhabdomyoma (55.3% vs. children/adolescents 61.4% and adults 24.5%) or lymphangioleiomyomatosis (10.6% vs. children/adolescents 0% and adults 11.5%).

There was also robust agreement between the two groups in terms of knowledge and assessment of individual disease manifestations. Good accordance could be found for epilepsy, one of the most relevant and life-affecting symptoms often leading to continuous medical treatment [21, 22]. There was also good agreement for cardiac rhabdomyomas and renal angiomyolipoma. The latter manifestation affects most adult patients and is associated with significant morbidity and mortality [23]. Interestingly, agreement on cutaneous manifestations differed markedly. Whereas the agreement for periungual fibroma was good for TSC- and not TSC-associated physicians, agreement for hypomelanotic macules was only good for TSC-associated physicians, while in the not TSC-associated group there was no correlation at all. This finding may be explained by the fact that several aforementioned symptoms can be assessed without technical devices and contribute to the burden of disease, whereas hypomelanotic macules are often difficult to detect and typically do no harm.

The moderate/poor correlation for retinal achromic patch in the total cohort might be because this manifestation is neither well known by patients nor by physicians, reflected by the high number of answers declared as ‘unknown’.

Additionally, the frequency of ‘unknown’ answers for certain manifestations is striking, particularly in relation to the compliance with monitoring recommendations. A physician’s declaration of an item as ‘unknown’ is likely to mean that no systematic assessment has been carried out for that manifestation.

Considering the physicians ‘answers, the highest number of ‘unknown’ answers was found for dental pits. This may indicate a lack of coordinating of dental care/assessment, which indeed can be challenging in patients with intellectual deficits. However, the fact that almost 30% of the corresponding patient questionnaires reported the presence of dental pits may also reflect a lack of careful and comprehensive physical examination (cf. Additional file 1: Table S2). The same applies for periungual fibroma (present according to patient answers in 41% of cases declared as ‘unknown’ by the physician), hypomelanotic macules (present according to patient answers in 71%) and fibrous cephalic plaque (present according to patient answers in 44%). It is alarming that 12% of the physicians declared the presence of renal angiomyolipoma as ‘unknown’, whereas 6% of the physicians declared the presence of SEGA as ‘unknown’, because along with epilepsy, these symptoms are major contributors to morbidity and mortality and regular assessment is mandatory. Regarding SEGA and considering a quite high number of ‘unknown’ answers for SEN, the finding may be explained by an uncertainty of diagnostic criteria for SEGA as stated above.

An ‘unknown’ answer on the patient’s part may be explained by a lack of patient education or may reflect individual coping strategies. The number of ‘unknown’ answers is much higher for physicians than for patients, possibly because patients are more likely to declare an item as not present (‘no’) although they do not know about it.

Strikingly, questionnaires completed by physicians who were associated with specialized TSC centre had a significantly lower number of items declared as ‘unknown’ compared to physicians not associated with TSC centres. Due to the complexity and highly diverse nature of TSC, a multidisciplinary approach in the management of the disease is recommended [8, 10].

It is known that a single centre establishment of a coordinated, multidisciplinary TSC board results in a significantly higher proportion of regular follow-up opportunities for patients to undergo examinations or to receive specialized treatments [24].

However, one limitation of this study is that only members of a patient advocacy group and their physicians were included. This might lead to a selection bias, as patients who are organized in a patient advocacy group might have more knowledge about the disease, might be more likely to be seen by specialists and might have a higher degree of disease severity. The response rate < 16% might also induce a bias with regard to a complete coverage of the contacted cohort. It is also worth mentioning that the TSC centres in Germany are certified by this patient organization.

## Conclusions

In conclusion we found a good agreement between the patient’s/caregiver’s and the physician’s assessment of those TSC manifestation known to be associated with relevant morbidity and mortality and of the overall estimation of disease severity. This suggests reasonable surveillance strategies, and sufficient patient/caregiver interaction and education in the examined cohort. Patients treated in specialized TSC centres appear to receive surveillance that is more comprehensive.

### Supplementary Information

**Additional file 1**. Flow chart of included questionnaires and Supplementary Tables.

## Data Availability

The datasets supporting the conclusions of this article are included within the article (and its additional files).
