# Supplementary material for: Patients’ and physicians’ awareness of clinical symptoms and disease severity in tuberous sclerosis complex
Source: Orphanet J Rare Dis. 2024 Mar 8;19:106. doi: 10.1186/s13023-024-03118-9 (PMC10921799; doi:10.1186/s13023-024-03118-9)
Supplement: Supplementary file 1 — Additional file 1. Flow chart of included questionnaires and Supplementary Tables. [file 13023_2024_3118_MOESM1_ESM.docx]

**Supplement**

**
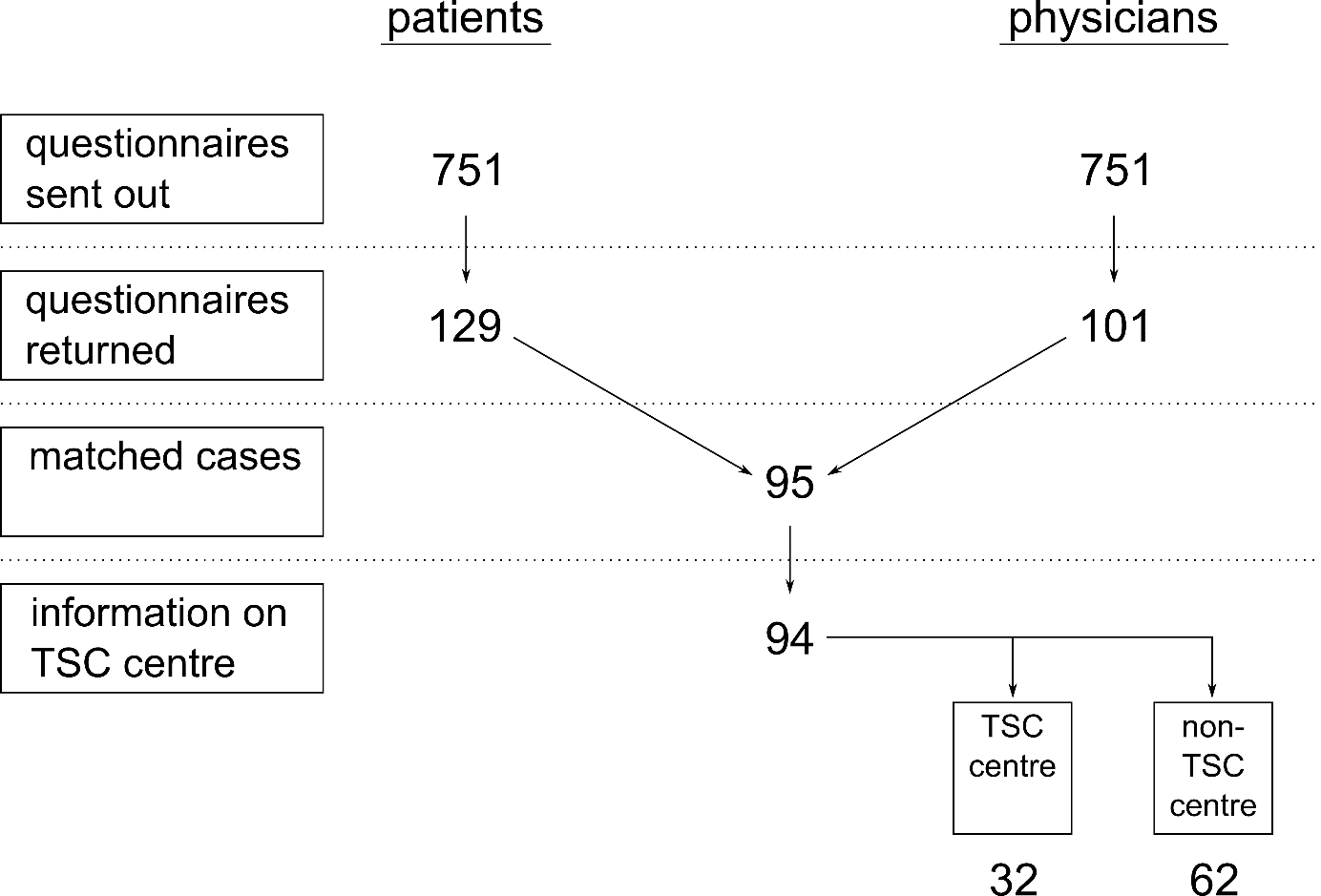
**

**Supplement Figure 1:** **Flow of the patients’ and doctors’ questionnaires used for the evaluation.**

Data are displayed in number and percentage of the relevant cohort (in parenthesis) if not otherwise declared. SEN: subependymale nodule, SEGA: subependymale giant cell astrocytoma, LAM: pulmonary lymphangioleiomyomatosis.

**Supplement Table S1: Demographic data and prevalence of disease manifestations in the respective groups.**

|  | | **Patient questionnaire** | **Physician questionnaire** |
| --- | --- | --- | --- |
| **Number** | | 94 | |
| **Sex** | | Male: 50 (53.2%),  Female: 44 (46.8%) | |
| **Age** | | Median: 18 years  [range 1-55] | |
| **Manifestations** | |  |  |
| skin | |  |  |
|  | hyomelanotic macules | 84 (89.36%) | 70 (74.47%) |
|  | facial angiofibroma | 80 (85.11%) | 66 (70.21%) |
|  | fibrous cephalic plaque | 37 (39.36%) | 25 (26.6%) |
|  | chagrin patch | 45 (47.87%) | 32 (34.04%) |
|  | periungual fibroma | 43 (45.74%) | 28 (29.79%) |
|  | fibroma pendulans | 24 (25.53%) | 15 (15.96%) |
|  | dental pits | 36 (38.3%) | 23 (24.47%) |
|  | gingival fibroma | 21 (22.34%) | 20 (21.28%) |
| eye | |  |  |
|  | retinal hamartoma | 23 (24.47%) | 18 (19.15%) |
|  | retinal achromatic patch | 6 (6.38%) | 3 (3.19%) |
|  | unilateral visual impairment | 5 (5.32%) | 6 (6.38%) |
|  | bilateral visual impairment | 3 (3.19%) | 2 (2.13%) |
|  | blindness | 1 (1.06%) | 2 (2.13%) |
| brain | |  |  |
|  | SEN | 74 (78.72%) | 66 (70.21%) |
|  | SEGA | 37 (39.36%) | 39 (41.49%) |
|  | tubera | 66 (70.21%) | 74 (78.72%) |
|  | dystopia | 12 (12.77%) | 18 (19.15%) |
|  | cerebral aneurysm | 1 (1.06%) | 2 (2.13%) |
|  | epilepsy | 84 (89.36%) | 80 (85.11%) |
|  | infantile spasms | 53 (56.38%) | 42 (44.68%) |
|  | status epilepticus | 25 (26.6%) | 22 (23.4%) |
|  | epilepsy surgery | 7 (7.45%) | 9 (9.57%) |
| neuropsychiatric disorders | |  |  |
|  | autism/autism spectrum disorder | 41 (43.62%) | 41 (43.62%) |
|  | aggressive behaviour | 32 (34.04%) | 26 (27.66%) |
|  | anxiety | 31 (32.98%) | 25 (26.6%) |
|  | compulsion | 33 (35.11%) | 17 (18.09%) |
|  | intellectual disability | 58 (61.7%) | 57 (60.64%) |
|  | non-verbal communication | 16 (17.02%) | 16 (17.02%) |
|  | unable to communicate | 10 (10.64%) | 5 (5.32%) |
| heart | |  |  |
|  | cardiac rhabdomyoma | 52 (55.32%) | 50 (53.19%) |
|  | effects on cardiac blood circulation by rhabdomyoma | 4 (4.26%) | 2 (2.13%) |
|  | arrhythmia by rhabdomyoma | 13 (13.83%) | 7 (7.45%) |
|  | aortic aneurysm | 2 (2.13%) | 2 (2.13%) |
| kidney | |  |  |
|  | renal angiomyolipoma | 54 (57.45%) | 54 (57.45%) |
|  | angiomyolipoma hemorrhage | 9 (9.57%) | 5 (5.32%) |
|  | renal cysts | 38 (40.43%) | 35 (37.23%) |
|  | renal insufficiency | 12 (12.77%) | 6 (6.38%) |
|  | renal cell carcinoma | 1 (1.06%) | 0 (0%) |
|  | dialysis-dependency | 2 (2.13%) | 2 (2.13%) |
| lung | |  |  |
|  | LAM | 10 (10.64%) | 14 (14.89%) |
|  | pneumothorax | 6 (6.38%) | 3 (3.19%) |
|  | chylothorax | 0 (0%) | 0 (0%) |
|  | ascites | 0 (0%) | 0 (0%) |
|  | oxygen feed | 1 (1.06%) | 0 (0%) |
| liver/GI/pancreas | |  |  |
|  | liver transplantation | 0 (0%) | 0 (0%) |
|  | colon polyp | 3 (3.19%) | 1 (1.06%) |
|  | Incontinence | 18 (19.15%) | 9 (9.57%) |
|  | hepatic angioymolipoma | 13 (13.83%) | 18 (19.15%) |
|  | pancreatic angiomyolipoma | 1 (1.06%) | 1 (1.06%) |
|  | pancreas tumor | 0 (0%) | 0 (0%) |
| locomotor system | |  |  |
|  | scoliosis | 24 (25.53%) | 17 (18.09%) |
|  | impaired ambulatory ability | 31 (32.98%) | 19 (20.21%) |
|  | unable to walk | 5 (5.32%) | 5 (5.32%) |

**Supplement Table 2: Cohen’s kappa coefficient representing the correlation between patient and physician questionnaires.**

|  | | patient  🡫 | physician | | | Cohen’s kappa coefficient | |
| --- | --- | --- | --- | --- | --- | --- | --- |
|  |  |  | no | yes | unknown | * | ** |
| Skin | |  |  |  |  |  |  |
|  | hypomelanotic macules | no | 2 | 1 | 4 | 0.364 | 0.21 |
|  |  | yes | 5 | 67 | 12 |  |  |
|  |  | unknown | 0 | 2 | 1 |  |  |
|  | facial angiofibroma | no | 12 | 1 | 0 | **0.613** | 0.482 |
|  |  | yes | 10 | 64 | 6 |  |  |
|  |  | unknown | 0 | 1 | 0 |  |  |
|  | fibrous cephalic plaque | no | 41 | 2 | 9 | **0.646** | 0.389 |
|  |  | yes | 10 | 20 | 7 |  |  |
|  |  | unknown | 2 | 3 | 0 |  |  |
|  | chagrin patch | no | 31 | 4 | 5 | 0.585 | 0.45 |
|  |  | yes | 11 | 26 | 8 |  |  |
|  |  | unknown | 2 | 2 | 5 |  |  |
|  | periungual fibroma | no | 36 | 0 | 13 | **0.799** | 0.464 |
|  |  | yes | 7 | 27 | 9 |  |  |
|  |  | unknown | 1 | 1 | 0 |  |  |
|  | fibroma pendulans | no | 40 | 1 | 15 | **0.647** | 0.232 |
|  |  | yes | 7 | 11 | 6 |  |  |
|  |  | unknown | 9 | 3 | 2 |  |  |
|  | dental pits | no | 26 | 5 | 23 | 0.499 | 0.245 |
|  |  | yes | 9 | 17 | 10 |  |  |
|  |  | unknown | 0 | 1 | 3 |  |  |
|  | gingival fibroma | no | 40 | 4 | 24 | **0.743** | 0.295 |
|  |  | yes | 2 | 13 | 6 |  |  |
|  |  | unknown | 1 | 3 | 1 |  |  |
| Eye | |  |  |  |  |  |  |
|  | retinal hamartoma | no | 43 | 2 | 16 | **0.794** | 0.393 |
|  |  | yes | 3 | 14 | 6 |  |  |
|  |  | unknown | 4 | 2 | 4 |  |  |
|  | retinal achromatic patch | no | 46 | 1 | 25 | 0.461 | 0.177 |
|  |  | yes | 3 | 2 | 1 |  |  |
|  |  | unknown | 7 | 0 | 9 |  |  |
|  | unilateral visual impairment | no | 57 | 3 | 21 | 0.568 | 0.168 |
|  |  | yes | 1 | 3 | 1 |  |  |
|  |  | unknown | 5 | 0 | 3 |  |  |
|  | bilateral visual impairment | no | 60 | 2 | 22 | -0.038 | 0.037 |
|  |  | yes | 3 | 0 | 0 |  |  |
|  |  | unknown | 4 | 0 | 3 |  |  |
|  | Blindness | no | 69 | 1 | 20 | 0.66 | 0.092 |
|  |  | yes | 0 | 1 | 0 |  |  |
|  |  | unknown | 2 | 0 | 1 |  |  |
| Brain | |  |  |  |  |  |  |
|  | SEN | no | 8 | 4 | 1 | 0.534 | 0.258 |
|  |  | yes | 6 | 56 | 12 |  |  |
|  |  | unknown | 0 | 6 | 1 |  |  |
|  | SEGA | no | 43 | 5 | 4 | **0.782** | **0.649** |
|  |  | yes | 4 | 32 | 1 |  |  |
|  |  | unknown | 2 | 2 | 1 |  |  |
|  | Tubera | no | 10 | 7 | 4 | **0.624** | 0.484 |
|  |  | yes | 2 | 62 | 2 |  |  |
|  |  | unknown | 0 | 5 | 2 |  |  |
|  | Dystopia | no | 37 | 7 | 14 | 0.469 | 0.215 |
|  |  | yes | 3 | 7 | 2 |  |  |
|  |  | unknown | 12 | 4 | 8 |  |  |
|  | cerebral aneurysm | no | 62 | 1 | 18 | **0.66** | 0.013 |
|  |  | yes | 0 | 1 | 0 |  |  |
|  |  | unknown | 10 | 0 | 2 |  |  |
|  | Epilepsy | no | 12 | 2 | 0 | **0.832** | **0.832** |
|  |  | yes | 2 | 78 | 0 |  |  |
|  |  | unknown | 0 | 0 | 0 |  |  |
|  | infantile spasms | no | 30 | 5 | 4 | **0.649** | 0.499 |
|  |  | yes | 9 | 36 | 8 |  |  |
|  |  | unknown | 0 | 1 | 1 |  |  |
|  | status epilepticus | no | 53 | 9 | 5 | 0.488 | 0.392 |
|  |  | yes | 7 | 13 | 5 |  |  |
|  |  | unknown | 1 | 0 | 1 |  |  |
|  | epilepsy surgery | no | 81 | 2 | 2 | **0.863** | **0.671** |
|  |  | yes | 0 | 7 | 0 |  |  |
|  |  | unknown | 2 | 0 | 0 |  |  |
| neuropsychological disorders | |  |  |  |  |  |  |
|  | autism/autism spectrum disorder | no | 39 | 7 | 6 | **0.673** | 0.565 |
|  |  | yes | 7 | 33 | 1 |  |  |
|  |  | unknown | 0 | 1 | 0 |  |  |
|  | aggressive behaviour | no | 49 | 6 | 6 | 0.556 | 0.45 |
|  |  | yes | 11 | 20 | 1 |  |  |
|  |  | unknown | 1 | 0 | 0 |  |  |
|  | Anxiety | no | 46 | 6 | 6 | **0.621** | 0.413 |
|  |  | yes | 7 | 19 | 5 |  |  |
|  |  | unknown | 5 | 0 | 0 |  |  |
|  | Compulsion | no | 45 | 3 | 11 | 0.539 | 0.317 |
|  |  | yes | 11 | 14 | 8 |  |  |
|  |  | unknown | 2 | 0 | 0 |  |  |
|  | intellectual disability | no | 29 | 5 | 1 | **0.786** | **0.718** |
|  |  | yes | 4 | 52 | 2 |  |  |
|  |  | unknown | 1 | 0 | 0 |  |  |
|  | non-verbal communication | no | 66 | 6 | 1 | 0.491 | 0.395 |
|  |  | yes | 7 | 9 | 0 |  |  |
|  |  | unknown | 4 | 1 | 0 |  |  |
|  | unable to communicate | no | 78 | 0 | 1 | **0.639** | 0.435 |
|  |  | yes | 5 | 5 | 0 |  |  |
|  |  | unknown | 5 | 0 | 0 |  |  |
| Heart | |  |  |  |  |  |  |
|  | cardiac rhabdomyoma | no | 27 | 2 | 7 | **0.865** | **0.639** |
|  |  | yes | 3 | 47 | 2 |  |  |
|  |  | unknown | 4 | 1 | 1 |  |  |
|  | effects on cardiac blood circulation by rhabdomyoma | no | 72 | 1 | 11 | 0.309 | 0.077 |
|  |  | yes | 3 | 1 | 0 |  |  |
|  |  | unknown | 5 | 0 | 1 |  |  |
|  | arrhythmia by rhabdomyoma | no | 63 | 2 | 12 | 0.465 | 0.234 |
|  |  | yes | 7 | 5 | 1 |  |  |
|  |  | unknown | 3 | 0 | 1 |  |  |
|  | aortic aneurysm | no | 72 | 0 | 15 | **1** | 0.239 |
|  |  | yes | 0 | 2 | 0 |  |  |
|  |  | unknown | 3 | 0 | 2 |  |  |
| Kidney | |  |  |  |  |  |  |
|  | renal angiomyolipoma | no | 25 | 3 | 5 | **0.807** | 0.596 |
|  |  | yes | 4 | 46 | 4 |  |  |
|  |  | unknown | 0 | 5 | 2 |  |  |
|  | angiomyolipoma hemorrhage | no | 66 | 1 | 12 | **0.705** | 0.21 |
|  |  | yes | 2 | 4 | 3 |  |  |
|  |  | unknown | 6 | 0 | 0 |  |  |
|  | renal cysts | no | 36 | 5 | 6 | **0.659** | 0.485 |
|  |  | yes | 8 | 28 | 2 |  |  |
|  |  | unknown | 5 | 2 | 2 |  |  |
|  | renal insufficiency | no | 68 | 0 | 10 | **0.674** | 0.359 |
|  |  | yes | 5 | 6 | 1 |  |  |
|  |  | unknown | 3 | 0 | 1 |  |  |
|  | renal cell carcinoma | no | 77 | 0 | 11 | *0** | 0.036 |
|  |  | yes | 1 | 0 | 0 |  |  |
|  |  | unknown | 4 | 0 | 1 |  |  |
|  | dialysis-dependency | no | 81 | 0 | 8 | 1 | 0.224 |
|  |  | yes | 0 | 2 | 0 |  |  |
|  |  | unknown | 3 | 0 | 0 |  |  |
| Lung | |  |  |  |  |  |  |
|  | LAM | no | 49 | 4 | 13 | **0.795** | 0.399 |
|  |  | yes | 0 | 10 | 0 |  |  |
|  |  | unknown | 11 | 0 | 7 |  |  |
|  | Pneumothorax | no | 61 | 0 | 12 | **0.735** | 0.277 |
|  |  | yes | 2 | 3 | 1 |  |  |
|  |  | unknown | 11 | 0 | 4 |  |  |
|  | chylothorax | no | 65 | 0 | 13 | *0** | 0.081 |
|  |  | yes | 0 | 0 | 0 |  |  |
|  |  | unknown | 12 | 0 | 4 |  |  |
|  | Ascites | no | 65 | 0 | 13 | *0** | 0.081 |
|  |  | yes | 0 | 0 | 0 |  |  |
|  |  | unknown | 12 | 0 | 4 |  |  |
|  | oxygen feed | no | 68 | 0 | 14 | *0** | 0.074 |
|  |  | yes | 1 | 0 | 0 |  |  |
|  |  | unknown | 8 | 0 | 3 |  |  |
| liver/GI/pancreas | |  |  |  |  |  |  |
|  | liver transplantation | no | 69 | 0 | 14 | *0** | 0.084 |
|  |  | yes | 0 | 0 | 0 |  |  |
|  |  | unknown | 8 | 0 | 3 |  |  |
|  | colon polyp | no | 48 | 0 | 16 | 0.485 | 0.3 |
|  |  | yes | 2 | 1 | 0 |  |  |
|  |  | unknown | 12 | 0 | 15 |  |  |
|  | Incontinence | no | 54 | 1 | 7 | 0.504 | 0.347 |
|  |  | yes | 8 | 6 | 4 |  |  |
|  |  | unknown | 7 | 2 | 5 |  |  |
|  | hepatic angiomyolipoma | no | 44 | 7 | 8 | **0.636** | 0.396 |
|  |  | yes | 1 | 10 | 2 |  |  |
|  |  | unknown | 12 | 1 | 9 |  |  |
|  | pancreatic angiomyolipoma | no | 58 | 1 | 11 | -0.017 | 0.262 |
|  |  | yes | 1 | 0 | 0 |  |  |
|  |  | unknown | 13 | 0 | 10 |  |  |
|  | pancreas tumor | no | 61 | 0 | 11 | *0** | 0.265 |
|  |  | yes | 0 | 0 | 0 |  |  |
|  |  | unknown | 13 | 0 | 9 |  |  |
| locomotor system | |  |  |  |  |  |  |
|  | Scoliosis | no | 54 | 5 | 5 | 0.385 | 0.265 |
|  |  | yes | 12 | 9 | 3 |  |  |
|  |  | unknown | 3 | 3 | 0 |  |  |
|  | impaired ambulatory ability | no | 45 | 2 | 10 | 0.536 | 0.338 |
|  |  | yes | 13 | 15 | 3 |  |  |
|  |  | unknown | 3 | 2 | 1 |  |  |
|  | unable to walk | no | 66 | 0 | 18 | **0.881** | 0.238 |
|  |  | yes | 1 | 4 | 0 |  |  |
|  |  | unknown | 3 | 1 | 1 |  |  |

Data are displayed as number and of the relevant cohort. ‘Yes’, ‘no’ and ´unknown´ answers were included in the analysis. Cohens kappa coefficient for the manifestation was calculated twice: On the one hand considering only ‘yes’ and ‘no’ answers (*) and on the other hand including the answers declared as unknown (**). Coefficients indicating moderate (0.60 – 0.79) or strong (>0.8) correlation are marked in bold letters whereas coefficients indicating none (0.00-0.20) correlation were marked in underlined letters. Cohen’s kappa statistics were based on the work of McHugh ^15^. SEN: subependymal nodule, SEGA: subependymal giant cell astrocytoma, LAM: pulmonary lymphangioleiomyomatosis

**Supplement Table 3: Number and proportion of disease manifestations declared as ‘unknown’ by patients and physicians.**

|  | | **Patient questionnaire**  **(n = 94)** | **Physician questionnaire** | | |
| --- | --- | --- | --- | --- | --- |
|  | |  | **total (n = 94)** | **TSC (n = 32)** | **non-TSC (n = 62)** |
| **all 53 symptoms** | | 435 (8.73 %) | 822 (16.50 %) | 147 (8.67 %) | 675 (20.54 %) |
| Skin | |  |  |  |  |
|  | hyomelanotic macules | 3 (3.19%) | 17 (18.09%) | 3 (9.38%) | 14 (22.58%) |
|  | facial Angiofibroma | 1 (1.06%) | 6 (6.38%) | 0 (0%) | 6 (9.68%) |
|  | fibrous cephalic plaque | 5 (5.32%) | 16 (17.02%) | 1 (3.13%) | 15 (24.19%) |
|  | chagrin patch | 9 (9.57%) | 18 (19.15%) | 2 (6.25%) | 16 (25.81%) |
|  | periungual fibroma | 2 (2.13%) | 22 (23.4%) | 1 (3.13%) | 21 (33.87%) |
|  | fibroma pendulans | 14 (14.89%) | 23 (24.47%) | 4 (12.5%) | 19 (30.65%) |
|  | dental pits | 4 (4.26%) | 36 (38.3%) | 8 (25%) | 28 (45.16%) |
|  | gingival fibroma | 5 (5.32%) | 31 (32.98%) | 6 (18.75%) | 25 (40.32%) |
| Eye | |  |  |  |  |
|  | retinal hamartoma | 10 (10.64%) | 26 (27.66%) | 5 (15.63%) | 21 (33.87%) |
|  | retinal achromatic patch | 16 (17.02%) | 35 (37.23%) | 7 (21.88%) | 28 (45.16%) |
|  | unilateral visual impairment | 8 (8.51%) | 25 (26.6%) | 5 (15.63%) | 20 (32.26%) |
|  | bilateral visual impairment | 7 (7.45%) | 25 (26.6%) | 5 (15.63%) | 20 (32.26%) |
|  | Blindness | 3 (3.19%) | 21 (22.34%) | 5 (15.63%) | 16 (25.81%) |
| Brain | |  |  |  |  |
|  | SEN | 7 (7.45%) | 14 (14.89%) | 1 (3.13%) | 13 (20.97%) |
|  | SEGA | 5 (5.32%) | 6 (6.38%) | 0 (0%) | 6 (9.68%) |
|  | Tubera | 7 (7.45%) | 8 (8.51%) | 0 (0%) | 8 (12.9%) |
|  | Dystopia | 24 (25.53%) | 24 (25.53%) | 3 (9.38%) | 21 (33.87%) |
|  | cerebral aneurysm | 12 (12.77%) | 20 (21.28%) | 1 (3.13%) | 19 (30.65%) |
|  | Epilepsy | 0 (0%) | 0 (0%) | 0 (0%) | 0 (0%) |
|  | infantile spasms | 2 (2.13%) | 13 (13.83%) | 6 (18.75%) | 7 (11.29%) |
|  | status epilepticus | 2 (2.13%) | 11 (11.7%) | 2 (6.25%) | 9 (14.52%) |
|  | epilepsy surgery | 2 (2.13%) | 2 (2.13%) | 0 (0%) | 2 (3.23%) |
| neuropsychological disorders | |  |  |  |  |
|  | autism/autism spectrum disorder | 1 (1.06%) | 7 (7.45%) | 4 (12.5%) | 3 (4.84%) |
|  | aggressive behaviour | 1 (1.06%) | 7 (7.45%) | 3 (9.38%) | 4 (6.45%) |
|  | Anxiety | 5 (5.32%) | 11 (11.7%) | 3 (9.38%) | 8 (12.9%) |
|  | Compulsion | 2 (2.13%) | 19 (20.21%) | 5 (15.63%) | 14 (22.58%) |
|  | intellectual disability | 1 (1.06%) | 3 (3.19%) | 2 (6.25%) | 1 (1.61%) |
|  | non-verbal communication | 5 (5.32%) | 1 (1.06%) | 1 (3.13%) | 0 (0%) |
|  | unable to communicate | 5 (5.32%) | 1 (1.06%) | 1 (3.13%) | 0 (0%) |
| Heart | |  |  |  |  |
|  | cardiac rhabdomyoma | 6 (6.38%) | 10 (10.64%) | 1 (3.13%) | 9 (14.52%) |
|  | effects on cardiac blood circulation by rhabdomyoma | 6 (6.38%) | 12 (12.77%) | 1 (3.13%) | 11 (17.74%) |
|  | arrhythmia by rhabdomyoma | 4 (4.26%) | 14 (14.89%) | 1 (3.13%) | 13 (20.97%) |
|  | aortic aneurysm | 5 (5.32%) | 17 (18.09%) | 1 (3.13%) | 16 (25.81%) |
| Kidney | |  |  |  |  |
|  | renal angioymolipoma | 7 (7.45%) | 11 (11.7%) | 0 (0%) | 11 (17.74%) |
|  | angiomyolipoma hemorrhage | 6 (6.38%) | 15 (15.96%) | 1 (3.13%) | 14 (22.58%) |
|  | renal cysts | 9 (9.57%) | 10 (10.64%) | 0 (0%) | 10 (16.13%) |
|  | renal insufficiency | 4 (4.26%) | 12 (12.77%) | 1 (3.13%) | 11 (17.74%) |
|  | renal cell carcinoma | 5 (5.32%) | 12 (12.77%) | 1 (3.13%) | 11 (17.74%) |
|  | dialysis-dependency | 3 (3.19%) | 8 (8.51%) | 1 (3.13%) | 7 (11.29%) |
| Lung | |  |  |  |  |
|  | LAM | 18 (19.15%) | 20 (21.28%) | 6 (18.75%) | 14 (22.58%) |
|  | Pneumothorax | 15 (15.96%) | 17 (18.09%) | 5 (15.63%) | 12 (19.35%) |
|  | chylothorax | 16 (17.02%) | 17 (18.09%) | 5 (15.63%) | 12 (19.35%) |
|  | Ascites | 16 (17.02%) | 17 (18.09%) | 5 (15.63%) | 12 (19.35%) |
|  | oxygen feed | 11 (11.7%) | 17 (18.09%) | 5 (15.63%) | 12 (19.35%) |
| liver/GI/pancreas | |  |  |  |  |
|  | liver transplantation | 11 (11.7%) | 17 (18.09%) | 5 (15.63%) | 12 (19.35%) |
|  | colon polyp | 27 (28.72%) | 31 (32.98%) | 6 (18.75%) | 25 (40.32%) |
|  | Incontinence | 14 (14.89%) | 16 (17.02%) | 2 (6.25%) | 14 (22.58%) |
|  | hepatic angioymolipoma | 22 (23.4%) | 19 (20.21%) | 2 (6.25%) | 17 (27.42%) |
|  | pancreatic angioymolipoma | 23 (24.47%) | 21 (22.34%) | 2 (6.25%) | 19 (30.65%) |
|  | pancreas tumor | 22 (23.4%) | 20 (21.28%) | 2 (6.25%) | 18 (29.03%) |
| locomotor system | |  |  |  |  |
|  | Scoliosis | 6 (6.38%) | 8 (8.51%) | 3 (9.38%) | 5 (8.06%) |
|  | impaired ambulatory ability | 6 (6.38%) | 14 (14.89%) | 4 (12.5%) | 10 (16.13%) |
|  | unable to walk | 5 (5.32%) | 19 (20.21%) | 3 (9.38%) | 16 (25.81%) |

The physicians’ answers are additionally shown after stratification by association to a TSC clinic.

**Supplement Table 4: Number and proportion of disease manifestations declared as ‘unknown’ by physicians** **after stratification by specialization.**

|  | | **Specialization** | | | | |
| --- | --- | --- | --- | --- | --- | --- |
|  | | **pediatrics (n = 20)** | **neuropediatrics (n = 31)** | **general medicine  (n = 15)** | **neurology  (n = 6)** | **nephrology  (n = 13)** |
| **all 53 symptoms** | | 193 (18.21 %) | 131 (7.97 %) | 188 (23.65 %) | 141 (44.34 %) | 86 (12.48 %) |
| Skin | |  |  |  |  |  |
|  | hyomelanotic macules | 3 (15 %) | 28 (6.45 %) | 5 (33.33 %) | 3 (50 %) | 2 (15.38 %) |
|  | facial Angiofibroma | 2 (10 %) | 15 (0 %) | 3 (20 %) | 1 (16.67 %) | 0 (0 %) |
|  | fibrous cephalic plaque | 4 (20 %) | 3 (3.23 %) | 4 (26.67 %) | 4 (66.67 %) | 2 (15.38 %) |
|  | chagrin patch | 4 (20 %) | 9 (3.23 %) | 5 (33.33 %) | 4 (66.67 %) | 2 (15.38 %) |
|  | periungual fibroma | 7 (35 %) | 4 (12.9 %) | 5 (33.33 %) | 3 (50 %) | 1 (7.69 %) |
|  | fibroma pendulans | 6 (30 %) | 0 (22.58 %) | 3 (20 %) | 5 (83.33 %) | 1 (7.69 %) |
|  | dental pits | 8 (40 %) | 7 (32.26 %) | 8 (53.33 %) | 6 (100 %) | 1 (7.69 %) |
|  | gingival fibroma | 10 (50 %) | 2 (22.58 %) | 6 (40 %) | 6 (100 %) | 1 (7.69 %) |
| Eye | |  |  |  |  |  |
|  | retinal hamartoma | 5 (25 %) | 5 (9.68 %) | 6 (40 %) | 4 (66.67 %) | 5 (38.46 %) |
|  | retinal achromatic patch | 8 (40 %) | 1 (19.35 %) | 7 (46.67 %) | 5 (83.33 %) | 5 (38.46 %) |
|  | unilateral visual impairment | 5 (25 %) | 1 (16.13 %) | 5 (33.33 %) | 4 (66.67 %) | 3 (23.08 %) |
|  | bilateral visual impairment | 5 (25 %) | 0 (16.13 %) | 5 (33.33 %) | 5 (83.33 %) | 3 (23.08 %) |
|  | Blindness | 3 (15 %) | 0 (12.9 %) | 4 (26.67 %) | 5 (83.33 %) | 3 (23.08 %) |
| Brain | |  |  |  |  |  |
|  | SEN | 4 (20 %) | 27 (0 %) | 4 (26.67 %) | 2 (33.33 %) | 1 (7.69 %) |
|  | SEGA | 2 (10 %) | 14 (0 %) | 3 (20 %) | 0 (0 %) | 0 (0 %) |
|  | Tubera | 2 (10 %) | 30 (0 %) | 3 (20 %) | 0 (0 %) | 0 (0 %) |
|  | Dystopia | 6 (30 %) | 8 (16.13 %) | 5 (33.33 %) | 1 (16.67 %) | 2 (15.38 %) |
|  | cerebral aneurysm | 6 (30 %) | 0 (6.45 %) | 5 (33.33 %) | 3 (50 %) | 0 (0 %) |
|  | Epilepsy | 0 (0 %) | 28 (0 %) | 0 (0 %) | 0 (0 %) | 0 (0 %) |
|  | infantile spasms | 5 (25 %) | 15 (3.23 %) | 1 (6.67 %) | 1 (16.67 %) | 5 (38.46 %) |
|  | status epilepticus | 3 (15 %) | 8 (6.45 %) | 1 (6.67 %) | 1 (16.67 %) | 2 (15.38 %) |
|  | epilepsy surgery | 0 (0 %) | 3 (0 %) | 1 (6.67 %) | 0 (0 %) | 0 (0 %) |
| neuropsychological disorders | |  |  |  |  |  |
|  | autism/autism spectrum disorder | 1 (5 %) | 14 (9.68 %) | 1 (6.67 %) | 1 (16.67 %) | 0 (0 %) |
|  | aggressive behaviour | 1 (5 %) | 8 (3.23 %) | 1 (6.67 %) | 2 (33.33 %) | 1 (7.69 %) |
|  | Anxiety | 3 (15 %) | 5 (6.45 %) | 1 (6.67 %) | 2 (33.33 %) | 1 (7.69 %) |
|  | Compulsion | 3 (15 %) | 5 (12.9 %) | 2 (13.33 %) | 6 (100 %) | 2 (15.38 %) |
|  | intellectual disability | 0 (0 %) | 18 (6.45 %) | 1 (6.67 %) | 0 (0 %) | 0 (0 %) |
|  | non-verbal communication | 0 (0 %) | 7 (3.23 %) | 0 (0 %) | 0 (0 %) | 0 (0 %) |
|  | unable to communicate | 0 (0 %) | 3 (3.23 %) | 0 (0 %) | 0 (0 %) | 0 (0 %) |
| Heart | |  |  |  |  |  |
|  | cardiac rhabdomyoma | 1 (5 %) | 24 (0 %) | 3 (20 %) | 3 (50 %) | 1 (7.69 %) |
|  | effects on cardiac blood circulation by rhabdomyoma | 3 (15 %) | 1 (0 %) | 3 (20 %) | 3 (50 %) | 1 (7.69 %) |
|  | arrhythmia by rhabdomyoma | 3 (15 %) | 1 (6.45 %) | 3 (20 %) | 3 (50 %) | 1 (7.69 %) |
|  | aortic aneurysm | 4 (20 %) | 1 (6.45 %) | 4 (26.67 %) | 4 (66.67 %) | 1 (7.69 %) |
| Kidney | |  |  |  |  |  |
|  | renal angioymolipoma | 4 (20 %) | 16 (0 %) | 5 (33.33 %) | 1 (16.67 %) | 0 (0 %) |
|  | angiomyolipoma hemorrhage | 3 (15 %) | 0 (3.23 %) | 6 (40 %) | 2 (33.33 %) | 2 (15.38 %) |
|  | renal cysts | 2 (10 %) | 9 (3.23 %) | 5 (33.33 %) | 1 (16.67 %) | 0 (0 %) |
|  | renal insufficiency | 2 (10 %) | 0 (3.23 %) | 5 (33.33 %) | 2 (33.33 %) | 1 (7.69 %) |
|  | renal cell carcinoma | 3 (15 %) | 0 (0 %) | 5 (33.33 %) | 1 (16.67 %) | 1 (7.69 %) |
|  | dialysis-dependency | 3 (15 %) | 0 (0 %) | 3 (20 %) | 1 (16.67 %) | 1 (7.69 %) |
| Lung | |  |  |  |  |  |
|  | LAM | 4 (20 %) | 2 (16.13 %) | 3 (20 %) | 4 (66.67 %) | 3 (23.08 %) |
|  | Pneumothorax | 5 (25 %) | 1 (9.68 %) | 2 (13.33 %) | 4 (66.67 %) | 2 (15.38 %) |
|  | chylothorax | 4 (20 %) | 0 (9.68 %) | 2 (13.33 %) | 4 (66.67 %) | 3 (23.08 %) |
|  | Ascites | 4 (20 %) | 0 (9.68 %) | 2 (13.33 %) | 4 (66.67 %) | 3 (23.08 %) |
|  | oxygen feed | 4 (20 %) | 0 (9.68 %) | 2 (13.33 %) | 4 (66.67 %) | 3 (23.08 %) |
| liver/GI/pancreas | |  |  |  |  |  |
|  | liver transplantation | 4 (20 %) | 0 (9.68 %) | 2 (13.33 %) | 4 (66.67 %) | 3 (23.08 %) |
|  | colon polyp | 9 (45 %) | 0 (16.13 %) | 6 (40 %) | 4 (66.67 %) | 4 (30.77 %) |
|  | Incontinence | 3 (15 %) | 3 (9.68 %) | 4 (26.67 %) | 3 (50 %) | 2 (15.38 %) |
|  | hepatic angioymolipoma | 3 (15 %) | 2 (9.68 %) | 6 (40 %) | 3 (50 %) | 2 (15.38 %) |
|  | pancreatic angioymolipoma | 4 (20 %) | 0 (9.68 %) | 6 (40 %) | 5 (83.33 %) | 2 (15.38 %) |
|  | pancreas tumor | 3 (15 %) | 0 (12.9 %) | 6 (40 %) | 5 (83.33 %) | 1 (7.69 %) |
| locomotor system | |  |  |  |  |  |
|  | Scoliosis | 2 (10 %) | 1 (0 %) | 2 (13.33 %) | 2 (33.33 %) | 2 (15.38 %) |
|  | impaired ambulatory ability | 4 (20 %) | 8 (9.68 %) | 3 (20 %) | 0 (0 %) | 2 (15.38 %) |
|  | unable to walk | 6 (30 %) | 3 (12.9 %) | 5 (33.33 %) | 0 (0 %) | 2 (15.38 %) |

**Supplement Table 5:** **Disease severity and degree of disability.**

| patients rating of disease severity | degree of disability | ECOG performance status | Barthel index |
| --- | --- | --- | --- |
| 9 | 100 | N/A | 95 |
| 1 | 0 | 1 | N/A |
| 3 | 0 | 1 | N/A |
| 5 | 90 | 1 | N/A |
| 1 | 0 | 1 | N/A |
| 2 | 0 | 1 | N/A |
| 1 | 0 | 1 | N/A |
| 4 | 50 | 1 | N/A |
| 7 | 0 | 1 | N/A |
| 3 | 80 | 1 | 65 |
| 6 | 100 | 1 | 65 |
| 6 | 90 | 1 | 75 |
| 4 | 0 | 1 | 95 |
| 3 | 0 | 1 | 100 |
| 6 | 50 | 1 | 100 |
| 2 | 70 | 1 | 100 |
| 1 | 0 | 1 | 100 |
| 2 | 0 | 1 | 100 |
| 5 | 50 | 1 | 100 |
| 4 | 80 | 1 | 100 |
| 7 | 80 | 1 | 100 |
| 3 | 0 | 1 | 100 |
| 5 | 0 | 1 | 100 |
| 4 | 0 | 1 | 100 |
| 1 | 100 | 1 | 100 |
| 4 | 90 | 1 | 100 |
| 5 | 80 | 1 | 100 |
| 4 | 0 | 1 | 100 |
| 2 | N/A | 1 | 100 |
| 4 | 100 | 1 | 100 |
| 3 | 50 | 1 | 100 |
| 1 | 0 | 1 | 100 |
| 4 | 70 | 1 | 100 |
| N/A | 40 | 1 | 100 |
| 5 | 90 | 1 | 100 |
| 3 | N/A | 1 | 100 |
| 8 | 100 | 2 | N/A |
| 8 | 70 | 2 | N/A |
| 8 | 100 | 2 | N/A |
| 5 | 100 | 2 | N/A |
| 7 | 50 | 2 | 40 |
| 5 | 70 | 2 | 50 |
| 4 | 80 | 2 | 50 |
| 3 | 70 | 2 | 65 |
| 7 | 100 | 2 | 75 |
| 5 | 90 | 2 | 80 |
| 7 | 100 | 2 | 80 |
| 4 | 100 | 2 | 80 |
| 4 | 90 | 2 | 85 |
| 7 | 80 | 2 | 85 |
| 5 | 100 | 2 | 100 |
| 7 | 100 | 2 | 100 |
| 7 | 70 | 2 | 100 |
| 6 | 60 | 2 | 100 |
| 6 | 50 | 2 | 100 |
| 3 | 70 | 2 | 100 |
| 1 | 100 | 2 | 100 |
| 3 | 60 | 2 | 100 |
| 3 | 100 | 2 | 100 |
| 3 | 50 | 3 | N/A |
| 9 | 100 | 3 | 30 |
| 7 | 100 | 3 | 65 |
| 8 | 100 | 3 | 65 |
| 8 | 100 | 3 | 75 |
| 7 | 70 | 3 | 75 |
| 7 | 100 | 3 | 90 |
| 7 | 100 | 3 | 90 |
| 7 | 50 | 3 | 100 |
| 8 | 100 | 4 | N/A |
| 10 | 100 | 4 | 20 |
| 6 | 100 | 4 | 60 |
| 7 | 100 | 5 | N/A |
| 10 | 100 | 5 | 0 |
| 7 | 100 | 5 | 0 |
| 7 | 100 | 5 | 10 |
| 9 | 100 | 5 | 10 |
| 7 | 100 | 5 | 15 |
| 9 | 100 | 5 | 25 |
| 10 | 100 | 5 | 30 |
| 9 | 100 | 5 | 30 |
| 9 | 100 | 5 | 30 |
| 6 | 90 | 5 | 35 |
| 8 | 100 | 5 | 35 |
| 7 | 100 | 5 | 45 |
| 8 | 100 | 5 | 50 |
| 5 | 100 | N/A | N/A |
| 5 | 100 | N/A | N/A |
| 10 | 100 | N/A | N/A |
| 6 | 0 | N/A | N/A |
| 6 | 70 | N/A | N/A |
| 5 | 100 | N/A | N/A |
| 6 | 80 | N/A | 0 |
| 9 | 100 | N/A | 20 |
| 8 | 80 | N/A | 75 |

Correlations were evaluated via Pearson correlation coefficient (PCC).
